# Representation of economic preferences in the structure and function of the amygdala and prefrontal cortex

**DOI:** 10.1038/srep20982

**Published:** 2016-02-15

**Authors:** Alan S. R. Fermin, Masamichi Sakagami, Toko Kiyonari, Yang Li, Yoshie Matsumoto, Toshio Yamagishi

**Affiliations:** 1 Graduate School of International Corporate Strategy, Hitotsubashi University, Tokyo, 101-8439 Japan; 2 Brain Science Institute, Tamagawa University, Machida, 194-8620 Japan; 3 School of Social Informatics, Aoyama Gakuin University, Sagamihara, 252-0206 Japan

## Abstract

Social value orientations (SVOs) are economic preferences for the distribution of resources – prosocial individuals are more cooperative and egalitarian than are proselfs. Despite the social and economic implications of SVOs, no systematic studies have examined their neural correlates. We investigated the amygdala and dorsolateral prefrontal cortex (DLPFC) structures and functions in prosocials and proselfs by functional magnetic resonance imaging and evaluated cooperative behavior in the Prisoner’s Dilemma game. We found for the first time that amygdala volume was larger in prosocials and positively correlated with cooperation, while DLPFC volume was larger in proselfs and negatively correlated with cooperation. Proselfs’ decisions were marked by strong DLPFC and weak amygdala activity, and prosocials’ decisions were marked by strong amygdala activity, with the DLPFC signal increasing only in defection. Our findings suggest that proselfs’ decisions are controlled by DLPFC-mediated deliberative processes, while prosocials’ decisions are initially guided by automatic amygdala processes.

In everyday life, humans experience social dilemmas regarding whether to follow social norms and cooperate with others at some personal cost or behave selfishly and maximize their own welfare. Social and economic studies have demonstrated that economic decisions are considerably influenced by individual differences in social value orientation (SVO)^1^,^2^,^3^,^4^,^5^,^6^, a social preference where individuals are classified as either prosocials or proselfs based on weights they assign to the distribution of resources between oneself and others^2^,^3^,^4^,^5^. Prosocials prefer a distribution of resources in which they and their partners jointly earn the most. In contrast, proselfs prefer the distribution that gives themselves the highest earnings, regardless of the partner’s payoff. SVO is consistently related to behavior in economic games^3^,^6^ and relates to self-sacrifice in real-life social relations^7^ as well as donation to charity^8^. Despite the strong implications of SVO on society, it has not yet been established whether these decisional dispositions have distinct structural and functional representations in the brain.

A wealth of behavioral evidence demonstrates that humans use distinct decision-making strategies for selfish and prosocial behaviors. Normative prosocial behaviors such as fairness, cooperation, spontaneous giving, and helping are increased by a number of factors that reduce deliberation, including the seriousness of social decisions^9^, cognitive load^10^, priming intuition^11^, and time pressure^12^,^13^. In addition, prosocial decisions occur significantly more quickly than selfish ones do^12^,^13^, while subjects make more selfish choices when a time delay is available for deliberation^12^,^14^. These findings suggest that humans may have an initial automatic impulse to behave prosocially that is sometimes overridden by deliberative processes necessary to implement selfish decisions.

Neuroscience studies support the existence of distinct neural networks for automatic and deliberative decision strategies in humans and animals^15^,^16^. Of special interest are the dorsolateral prefrontal cortex (DLPFC) and the amygdala. The role of the DLPFC has been demonstrated in the control of deliberative behaviors such as strategic decision-making^17^, inference, and reasoning^18^,^19^. On the other hand, the amygdala has been implicated in the control of automatic behaviors such as the expression of innate responses^20^, the acquisition of conditioned reactions to biologically significant stimuli^21^, and has recently been implicated in automatic social decision processes^22^,^23^,^24^.

We hypothesize that the DLPFC and amygdala are candidate regions underlying the respective deliberative and automatic processes of social decisions, and that their contributions depend on individual differences in SVO. More specifically, because DLPFC functions and selfish decisions have been associated with deliberation, and proselfs are predominantly selfish decision-makers, our hypothesis is that proselfs recruit DLPFC-mediated deliberative functions more than prosocials do. Conversely, because amygdala functions and prosocial behaviors have been associated with automaticity, and prosocials are predominantly cooperative, we hypothesize that prosocials recruit amygdala-mediated automatic functions more than proselfs do.

In order to test these hypotheses and identify differences in brain structure and function between prosocials and proselfs, we conducted voxel-based morphometry (VBM) and functional magnetic resonance imaging (fMRI) analyses on human subjects, and examined the relationship between these data with SVO and cooperative behavior in a sequential one-shot Prisoner’s Dilemma game (PDG; Fig. 1). In the sequential PDG used in this study, the subject was always the first player to make a choice, which was observed and followed by the partner’s decision. In the PDG, the players have the choice to either cooperate or defect, and their payoffs depend on the combination of their choices. Unilateral defection results in the highest payoff for the defector and nothing for the cooperator, while the payoff for mutual cooperation is higher than that for mutual defection. The PDG is a reliable experimental paradigm to study the conflict between the selfish choice to defect to maximize one’s own gain and the prosocial choice to cooperate and run the risk of being exploited by others. The rational choice for both players is to defect because, regardless of the partner’s choice, defection maximizes one’s own payoff. However, if individuals have predispositions to behave in a selfish or cooperative manner, then significant differences should be expected in choice behavior, with proselfs defecting more than prosocials.

Previous studies found a strong correlation between SVO and choice behavior in the PDG^2^,^3^,^4^,^5^,^6^,^7^, with a significantly higher defection rate among proselfs and a higher cooperation rate among prosocials, suggesting that the PDG is an ideal laboratory game for the study of individual economic preferences. Here, we show for the first time that amygdala volume is larger in prosocials and positively correlates with cooperation, while DLPFC volume is larger in proselfs and negatively correlates with cooperation. We also found stronger DLPFC activity and a weak amygdala signal in proselfs’ decisions, regardless of choice type. Conversely, amygdala activity was stronger in prosocials, but it was accompanied by DLPFC activity only in defection.

## Results

### Choice behavior in the Prisoner’s Dilemma game

Subjects were classified as either prosocials (n = 15) or proselfs (n = 18) based on the consistency of their choices in a one-shot PDG and in two tests of SVO^3^,^6^ (see Supplementary Methods for details on SVO classification). We analyzed subjects’ behavior while playing the sequential PDG inside the fMRI scanner. Error trials (12 of 990 trials, 0.01%) in which subjects did not push the button within the 12-s response period were excluded from this analysis. A generalized linear mixed model analysis of the binary decision of cooperation/defection, with SVO (prosocials and proselfs) and stake size (JPY 100, JPY 200, JPY 400) as fixed effects and the subject as a random effect, revealed that the mean cooperation level of the prosocials was higher than that of the proselfs was, and that overall cooperation rate increased as stake size became smaller. The first player’s cooperation rate is known to be higher than that typically observed in the simultaneous game using the same design^25^. Even prosocials in the role of the first player cooperated at a high level when the stake was small, although they cooperated at a much lower level when the stake was large. The lack of a significant SVO × stake size interaction revealed that despite the modulatory effect of stake size on choice behavior leading to less cooperation when the stake size was larger, a higher cooperation rate among prosocials as compared to proselfs remained present (Fig. 2b).

### Amygdala and DLPFC volumes represent social value orientation

We investigated whether brain structure has any relationship with SVO and cooperative behavior. We hypothesized that proselfs have larger DLPFC volume than prosocials do, whereas prosocials have larger amygdala volume than proselfs do. We tested this hypothesis by conducting a structural VBM analysis of subjects’ brains with a multiple regression using mask images of the amygdala and DLPFC (see Supplementary Methods for details). The regressors used in this analysis consisted of each subject’s average cooperation rate in the PDG and SVO classification tests (Supplementary Methods). Positive correlations with SVO were interpreted as regions with larger gray matter (GM) volume in prosocials, whereas negative correlations with SVO were identified as regions with larger GM volume in proselfs.

The VBM analysis revealed that the GM volume of the left amygdala (Fig. 3a) was significantly larger in prosocials than it was in proselfs (P < 0.05, familywise error [FWE] corrected with small volume correction defined by the mask image). The left amygdala volume also positively correlated with cooperation rate (P < 0.05 FWE corrected, Fig. 3b,c). Conversely, right DLPFC volume was larger in proselfs than it was in prosocials (P < 0.05 FWE corrected, Fig. 3d) and left DLPFC volume negatively correlated with cooperation rate (P < 0.05 FWE corrected, Fig. 3e,f). We confirmed these results using anatomical mask images of the amygdala and DLPFC (Supplementary Fig. 1). A whole-brain analysis demonstrated further structural heterogeneity between prosocials and proselfs (Supplementary Figs 2–5 and Supplementary Tables 1–4).

### The amygdala and DLPFC show SVO-dependent activity

We investigated whether the amygdala and DLPFC showed differential SVO-dependent activity. We hypothesized that proselfs’ decisions (which are selfish) are controlled by deliberative processes implemented in the DLPFC, and that prosocials’ decisions (which are predominantly cooperative and egalitarian) are controlled by automatic processes implemented in the amygdala. To test this hypothesis we used the voxel clusters in the left amygdala and left DLPFC, which showed positive and negative correlations, respectively, with cooperation rate in the VBM analysis. The activity in the right amygdala and right DLPFC was estimated after creating mask images by reversing the sign of the x-coordinate of the left amygdala and left DLPFC.

Our analysis focused on the 4–6-s delay between the display of stake size and the start of the response period. First, we examined differences in BOLD signal between prosocials and proselfs, regardless of choice type. The analysis found a significantly higher BOLD signal in proselfs compared to prosocials in the left DLPFC (F_(1,31)_ = 57.5, P < 0.0001) and right DLPFC (F_(1,31)_ = 29, P < 0.0001) (Fig. 4a). In contrast, the BOLD signal in the amygdala was stronger in prosocials compared to proselfs in both the left (F_(1,31)_ = 45.48, P < 0.0001) and right (F_(1,31)_ = 15.52, P = 0.0004) hemispheres (Fig. 4a).

Next, we analyzed the activity in the amygdala and DLPFC during the delay-period separated by choice type and SVO. This analysis revealed no significant differences between defection and cooperation choices among proselfs in the activity of the left (F_(1,33)_ = 0.97, P = 0.33) or right DLPFC (F_(1,33)_ = 0.16, P = 0.69) (Fig. 4b), or in the left (F_(1,33)_ = 0.02, P = 0.89) or right amygdala (F_(1,33)_ = 0.2, P = 0.65) (Fig. 4b). In prosocials, however, we found significantly higher activity in the left and right DLPFC (F_(1,23)_ = 4.34, P = 0.048; F_(1,23)_ = 4.54, P = 0.044, respectively) for defection compared to cooperation trials (Fig. 4b). No significant differences between defection and cooperation choices were found among prosocials in the left or right amygdala (F_(1,23)_ = 2.42, P = 0.13 and F_(1,23)_ = 3.41, P = 0.077, respectively; see Fig. 4b).

Neuroimaging and electrophysiological studies with humans have demonstrated that the automatic amygdala activity in response to social cues appears fast and precedes top-down DLPFC control^26^,^27^. Therefore, we estimated the time course of the amygdala and DLPFC BOLD signal during the delay to search for possible neural correlates indicative of automatic and deliberative processes in economic decision-making. This analysis revealed that, in proselfs, DLPFC activity increased early and was present throughout the delay (Fig. 5a), while no increase in amygdala signal was observed (Fig. 5b). Contrastingly, amygdala activity in prosocials appeared early and remained high throughout the delay (Fig. 5b), whereas DLPFC activity appeared late and only in defection, not in cooperation (Fig. 5a).

## Discussion

The higher cooperation rate among prosocials than among proselfs replicates previous SVO findings^3^,^4^,^5^ and validates the PDG as an experimental tool to investigate the neural basis of SVO. The left amygdala GM volume was larger in prosocials and positively correlated with cooperation rate. Contrastingly, the right DLPFC GM volume was larger in proselfs, and the left DLPFC GM volume negatively correlated with cooperation rate. The neuroimaging analysis found stronger DLPFC activity and weak amygdala signal among proselfs relative to prosocials, regardless of choice type. Conversely, amygdala activity was stronger in prosocials but was accompanied by DLPFC activity only in defection choices. These results demonstrate for the first time a neurobiological dissociation between individuals with cooperative and selfish economic preferences.

The variability of GM volumes in the amygdala and DLPFC associated with SVO and cooperative behavior is consistent with previous human neuroimaging studies showing a strong relationship between regional brain structures and social cognitive processes such as empathy^28^ and altruistic behavior^29^. Furthermore, amygdala volume is strongly related with social network size^30^ and DLPFC GM thickness with deliberative decision-making^31^. Our findings are also in line with the suggestion that the enlargement of the human brain compared to other primates and specifically of structures such as the amygdala and prefrontal cortex enabled humans to acquire cognitive skills to cope with social life^32^. To our knowledge, the current study is the first to find a direct relationship between prosocial behavior and the structures of the amygdala and DLPFC.

Genetic processes may play important roles in the heterogeneity of amygdala and DLPFC GM volumes as well as in SVO. Genetic studies have linked genes to the heritability of the overall GM volume in humans^33^ and also to distinct brain areas such as the amygdala^34^ and prefrontal cortex^35^. Twin studies also support the heritability of personality traits, for instance, more cooperative or selfish dispositions are observed in monozygotic than in dizygotic twins^36^. Genetic factors, however, only explain 50% of personality heritability^36^, suggesting an important role for nurture in personality formation and possibly in SVO, as well as in shaping brain structure.

Differences in SVO and brain structure may also reflect experience-dependent processes. For instance, the acquisition of prosociality in children is modulated by praising an infant’s character^37^ or observing an adult’s behavior^38^. Likewise, the GM volume of brain regions such as the DLPFC and amygdala is influenced by socioeconomic status^39^. A neuroimaging study with monkeys^40^ provides direct evidence of the effect of social environment on brain plasticity. The study found that, after placing monkeys in social groups of different sizes, measures of social network size, social rank, and dominance significantly correlated with increases in GM volume of regions involved in deliberation and social cognition, such as the prefrontal and temporal cortices and the amygdala. These studies suggest that the amygdala and DLPFC are susceptible to social-dependent neural plasticity, which may be influenced by the use of distinct social strategies such as seeking to reduce economic inequality or to maximize one’s own benefits.

The fMRI analysis revealed stronger DLPFC activity and weaker amygdala signal in proselfs than in prosocials (Fig. 4a). Computational and experimental studies implicate the DLPFC in deliberative decision-making using internal representations and contextual variables to calculate future outcomes of available choices^15^,^16^,^17^,^18^,^19^. The activation of the DLPFC by proselfs in both defection and cooperation suggests that proselfs use deliberation regardless of choice behavior (Fig. 4b). This interpretation is consistent with behavioral studies showing that proselfs deliberately maximize their payoffs and choose to either cooperate or defect whenever expecting economic benefits^3^,^4^,^5^,^6^. Neuroeconomic studies demonstrate that the DLPFC plays an important role in deliberative decision-making, such as in strategic norm compliance^41^, dishonest choices^42^ and strategic deception^43^ to maximize one’s own payoff. Furthermore, increasing DLPFC excitability increases selfish choices and punishment avoidance in order to maximize gains in the ultimatum game^44^. We suggest that proselfs continuously use deliberation, implemented in the DLPFC, to strategically pursue their self-interests, either by selfish or norm-abiding behaviors, estimating future outcomes resulting from the combination of one’s own and other’s likely choices.

Prosocials, contrary to proselfs, activated the DLPFC in defection but not in cooperation (Fig. 4b). In the PDG, defection is the selfish choice that maximizes one’s outcome at the expense of others. Therefore, a straightforward interpretation of our finding is that prosocials recruit the DLPFC to deliberately pursue selfish goals in the same manner as proselfs. However, the DLPFC is also activated when humans face moral dilemmas and violate social norms^45^. The activation of the DLPFC by prosocials only in defection, and the findings showing that they attribute choices in the PDG to morality concerns and associate cooperation and defection with good and bad behavior, respectively^46^, suggests that prosocials and proselfs may use deliberation for different purposes. Thus, an alternative explanation is that consideration to defect may elicit in prosocials a moral conflict between the choices to defect and cause social harm and the choice to follow social norms and cooperate with others. Prosocials would then recruit the DLPFC and employ deliberative processes to support conflict resolution and weigh the impact of selfish decisions on social welfare.

The structural and functional MRI analyses demonstrated a strong relationship between amygdala GM volume and functional activity with the disposition to cooperate (Fig. 3a–c) and prosocial decision-making (Fig. 4a,b). Clinical studies have also demonstrated that amygdala damage causes deficits in multiple aspects of social behavior such as diminished motivation to engage in interdependent relations^47^, poor judgment of trustworthiness^48^, and repeated cooperation with untrustworthy others^26^. One significant implication of these studies is that the amygdala may play an important role in the evaluation of social signals and such evaluation might be used to support behavior switching to avoid socially aversive outcomes, an interpretation consistent with the SVO literature demonstrating that prosocials switch from cooperation to defection in the PDG if expecting others to defect^3^,^4^.

In both humans and animals, amygdala function is linked with automatic control of innate and learned behaviors such as fear and Pavlovian conditioning^20^,^21^,^22^,^23^,^24^. Electroencephalography and electrophysiological recording studies with humans demonstrated that automatic amygdala signaling appears relatively fast in response to visual social cues and precedes deliberative control by the prefrontal cortex^27^,^49^. Analysis of the time course of the BOLD signal revealed similar activity patterns only in prosocials and showed that amygdala activity preceded that of the DLPFC, which appeared late in the decision period and only in defection choices (Fig. 5a,b). Our finding that prosocials activated the amygdala but not the DLPFC in cooperation (Fig. 4b) supports the hypothesis that prosocial decisions are first guided by automatic control^9^,^12^,^13^,^14^. The amygdala activation in cooperation in prosocials but not in proselfs further suggests that the automatic control of prosociality is dependent on differences in SVO and not simply on the distinction between prosocial and selfish decisions. Our findings are also supported by a study investigating the neural basis of SVO, which found a stronger amygdala signal in prosocials in a task requiring the evaluation of the distribution of payoffs for the self and a partner, suggesting that this signal represents an automatic decision process^23^.

We cannot rule out a possible interaction of the amygdala with other brain regions involved in automatic processes that could play a role in prosociality. For instance, the striatum is also implicated in habit formation through action value learning^15^,^16^,^18^, and neuroimaging studies suggest that the amygdala modulates the activity of the striatum^50^ to facilitate reward or aversive learning^51^. We might speculate that the amygdala and striatum contribute to the formation of prosocial habits in two stages: the amygdala uses a Pavlovian control mechanism to associate social cues with the valence of outcomes resulting from expected social interactions, whereas the striatum learns the expected values of classes of behaviors (e.g., cooperation) or specific actions that maximize social rewards (e.g., approach to collect social rewards, or escape to avoid harmful social encounters). The memory properties of the amygdala and striatum may be used not only in on-line evaluation of social cues and action values, but also to emulate the quality of future social interactions and selection of actions found to be successful or avoidance of actions found to be detrimental in previous experiences.

In summary, our results demonstrate a dissociation between prosocials and proselfs based on their economic choices and the structures and activities of the amygdala and DLPFC. Our findings also demonstrate that the participation of the amygdala and DLPFC in automatic and deliberative decision processes is dependent on SVO. The present findings suggest that economic studies should focus not only on whether different social choices (e.g., cooperation vs. defection, fair vs. unfair) could be under the control of distinct psychological processes, but also on how individual differences in economic preferences play a role in decision-making. Future research combining longitudinal and developmental approaches, genetic mapping, socioeconomic, and neuroimaging data may provide a significant contribution to the understanding of how genetic, social, and neural factors interact and operate in social decision-making.

## Methods

### Subjects

Participants were recruited from another university, Aoyama Gakuin University, where one of the authors (TK) works. Neurologically health university students (n = 41) were recruited to play a sequential non-matrix Prisoner’s Dilemma game (PDG) against human partners in functional magnetic resonance imaging (fMRI) experiment. Eight participants were excluded from further analysis due to brain abnormalities, large head movements inside the fMRI scanner, medication treatment, or erroneous behavior. The remaining 33 subjects were classified into two groups: the prosocial group (n = 15, 8 women, 20–22 years old, mean ± SD: 20.8, 0.5 years) and the proself group (n = 18, 10 women, 20–23 years old, mean ± SD: 21.1, 0.8 years). Experimental protocols were approved by the Ethics Committee of the Institute of Brain Science, Tamagawa University, and they meet the Declaration of Helsinki requirements. The methods were carried out in accordance to approved guidelines. All subjects signed an approved consent form.

### Assessment of social value orientation

The social value orientation (SVO) assessment for the classification of a subject as prosocial or proself was done by way of two methods: the triple-dominance method^52^ and the ring method^5^, in combination with the consistency of the subject’s choices in a one-shot PDG. First, subjects played a one-shot PDG and had to make either a cooperative or a selfish choice to exchange money with an anonymous partner. Next, subjects were assessed with the triple-dominance and ring methods. In the triple-dominance method, subjects are tested in nine items, each with three alternatives for the distribution of points for the self and an anonymous partner. In the ring method, subjects were tested with 24 items, each with two alternatives for the distribution of points for the self and partner. In both methods, subjects are classified as either prosocial or proself if they consistently choose the same distribution of points for the self and a partner in at least 60% of the items. The subjects were re-tested in the same two SVO measures after the fMRI study. Finally, the subjects whose choices in the PDG were consistent with their choice behavior in the two measures of SVO were classified as prosocials or proselfs. Subjects were re-tested with the one-shot PDG and SVO measures after the fMRI experiment. At the end of the fMRI experiment, subjects were paid a show-up fee of JPY 5000 (~USD 42) added to the sum of 10 randomly chosen actual payoff outcomes in the PDG.

### Experimental design

On the day of the fMRI study, subjects first underwent an fMRI scanning session for the acquisition of structural brain data. Subjects then moved to an experimental room for a computer-based instruction followed by a quiz. The experimental room had 10 small private booths with computer stations. Six subjects occupied the booths simultaneously, and none of them knew or spoke to each other. After the instruction and quiz, it was announced that one of the participants was going to be randomly assigned to play the PDG inside the fMRI scanner against the other participants, who remained in their respective booths. This procedure was employed to ensure ecological validity for social interaction between participants while playing the PDG.

**Figure 1:**
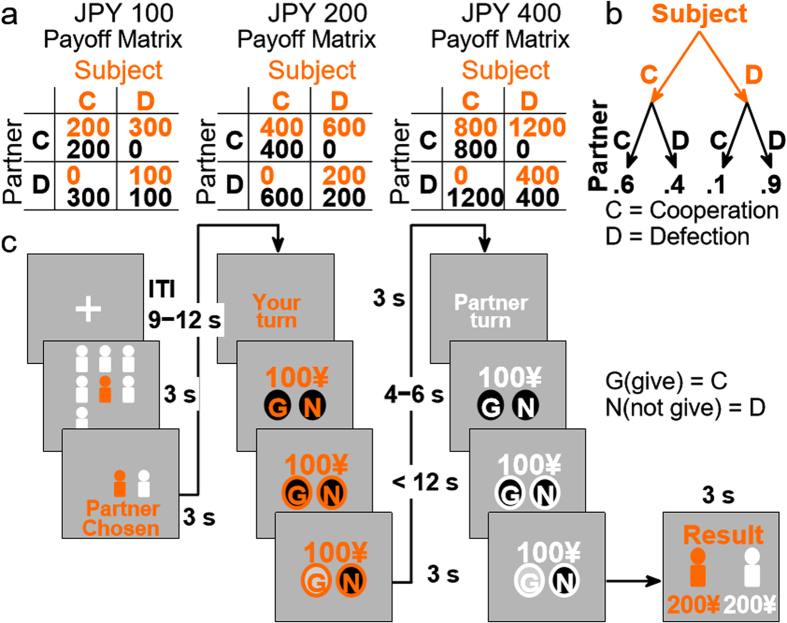
Experimental design and task diagram. (**a**) Stake size used in the sequential one-shot Prisoner’s Dilemma game and reconstructed payoff matrices. The payoff matrices themselves were not shown to subjects. (**b**) Player 2 preprogrammed the conditional choice probability based on the preceding choice of Player 1. (**c**) Task events. Following the inter-trial interval (ITI), each trial started with the random selection of an anonymous partner for the role of Player 2. The subject inside the fMRI scanner played the role of Player 1 and was the first to make a choice. Following the indication of whose turn it was to make a choice, a pseudo-randomly chosen stake size was displayed for 4–6 s. The subject was allowed to make a button press only during the response period (~12 s) indicated by a go signal (circle displayed around the buttons). A choice made within the response period highlighted the chosen button, while failing to make a choice displayed a failure message, although subjects were still requested to press a button. Player 1’s choice was observed and followed by the choice of Player 2. Both players had the choice to either Provide (G, cooperate) and transfer to the partner the whole stake, which was doubled in value, or Not Provide (N, defect) and keep the original stake value. Feedback, displayed at the end of every trial, showed the earned payoff by each player.

**Figure 2:**
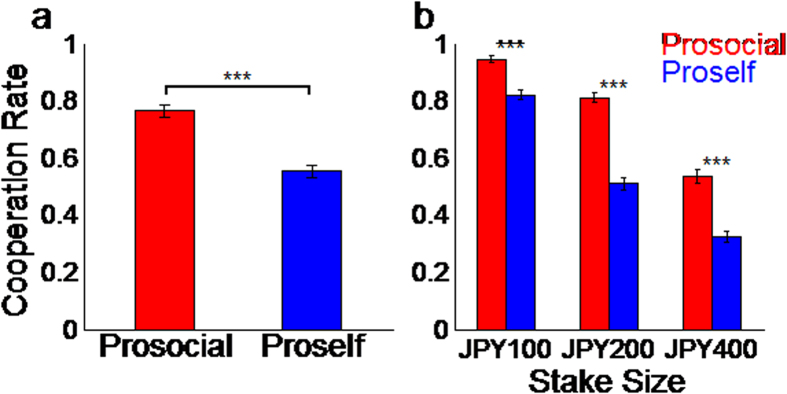
Choice behavior in the Prisoner’s Dilemma game. (**a**) The overall cooperation rate of prosocials was significantly higher compared to that of proselfs (F_(1,32)_ = 47.19, P < 0.0001). (**b**) Prosocials’ cooperation rate was also higher for each stake size. The main effect of stake size was significant (F_(2,64)_ = 63.35, P < 0.0001), indicating that cooperation rate decreased as the stake size increased. The social value orientation × stake size interaction was not significant (F_(2,64)_ = 1.65, P = 0.200), suggesting that prosocials’ cooperation was consistently higher than that of proselfs was, regardless of stake size.

**Figure 3:**
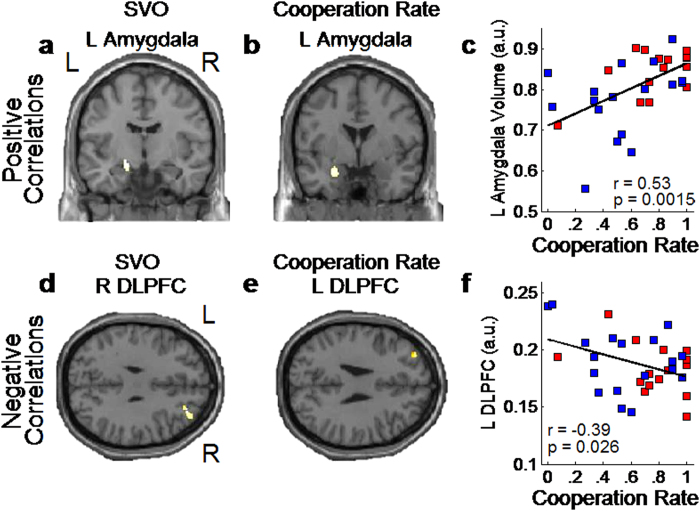
Correlation between amygdala and dorsolateral prefrontal cortex (DLPFC) gray matter volumes with social value orientation (SVO) and cooperative behavior in the Prisoner’s Dilemma game. (**a**) Left amygdala volume was significantly larger in prosocials than it was in proselfs (positive correlation with SVO, 66 voxels, x = −17, y = −9, z = −12, t = 2.61, P < 0.05 family-wise error (FWE) corrected) and (**b,c**) positively correlated with cooperation rate (81 voxels, x = −24, y = 0, z = −21, t = 2.57, P < 0.05 FWE corrected). (**d**) Right DLPFC volume was significantly larger in proselfs than it was in prosocials (negative correlation with SVO, 118 voxels, x = 29, y = 41, z = 30, t = 3.28, P < 0.05 FWE corrected), and (**e,f**) negatively correlated with cooperation rate (65 voxels, x = −30, y = 48, z = 33, t = 2.77, P < 0.05 FWE corrected). In (**c,f**), the regression line was computed for the whole sample; the red and blue dots represent individual data points of prosocials and proselfs, respectively. For visualization purposes, panels **(a–d**) and the voxel clusters are displayed at P < 0.001, uncorrected.

**Figure 4:**
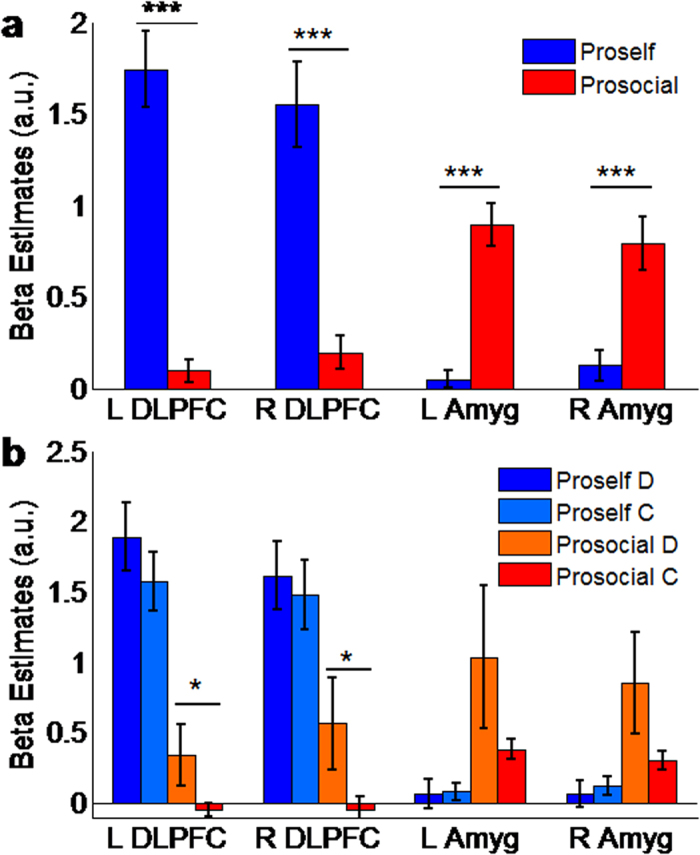
Activity signals (represented as beta weights) in the dorsolateral prefrontal cortex (DLPFC) and amygdala distinguish between prosocials and proselfs. (**a**) Overall signal intensity (beta estimates) in the DLPFC and amygdala during the delay period. (**b**) Signal intensity in the delay period separated by social value orientation and choice type. The stars on top of each bar set represent statistically significant differences. For (**a**), significant differences are between prosocials and proselfs in the amygdala (*** = P < 0.0001). For (**b**), significant differences are between choice types by prosocials in the DLPFC (* = P < 0.05). The beta parameters were estimated using the voxel clusters identified in the VBM analysis shown in Fig. 2.

**Figure 5:**
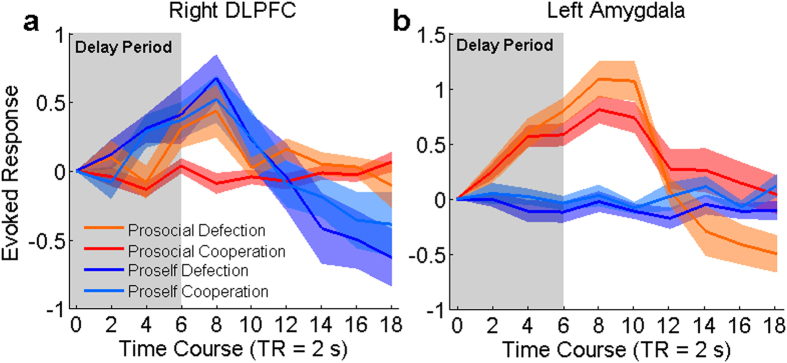
Time course of neural activity in the right dorsolateral prefrontal cortex (DLPFC) and left amygdala. (**a**) An early increase in signal was observed in the right DLPFC of proselfs when choosing to either cooperate or defect; in contrast, only a late signal increase was observed in prosocials for defection choices. (**b**) An early signal increase was observed in the left amygdala during both cooperative and defection choices by prosocials. No significant signal increase in the left amygdala was observed in proselfs, regardless of choice type. See the Methods section for more details regarding analysis.

The subject assigned for the fMRI scanning session played a sequential iterated one-shot PDG where the subject and the partners alternated the order of making their choices, that is, the first-player condition or second-player condition, in blocks of 15 trials and two blocks per condition. The order of player condition was counterbalanced across subjects. In this paper, we report the results of subjects’ choice behavior and fMRI data only in the first-player condition. One of three stake offers—JPY 100 (~USD 1), JPY 200 (~USD 2), and JPY 400 (~USD 4)—was randomly selected for each trial. Although no payoff matrix was shown in the current experiment, subjects could easily recreate it in their minds based on the rules of the game taught in the instruction session. In the first-player condition, the subject inside the fMRI scanner was the first to choose to either Provide (G) or Not Provide (N) (Fig. 1) the stake offer to the partner, which represented the choices to cooperate or defect, respectively. If the subject chose Provide (cooperate), the partner received double the value of the original stake offer (2 × JPY 100 = JPY 200), whereas the subject kept for himself/herself only the original stake offer if he/she chose Not Provide (defect).

Although the subject believed that he/she was playing against real human partners who remained in the instruction room, the partners’ choices were actually computer programmed using conditional probability. The partner cooperated (choice Provide) with a probability of 0.6 following a subject’s cooperation, and defected (choice Not Provide) with a probability of 0.9 following a subject’s defection. These probabilities were chosen to match actual human choice behavior in a one-shot non-repeated two-person PDG as documented in the literature. In addition, this conditional probability rule for the partner’s choice was chosen in order to equate the expected payoff value if a subject decided to only cooperate or defect. Therefore, the experimental design did not have any intrinsic constraints that could motivate subjects to assume unconditional cooperative or selfish decision strategies in order to maximize their payoff.

In order to test the efficacy of this experimental design to control for expected payoff, we performed two separate one-way ANOVAs to investigate differences in average payoff (estimated separately for prosocials and proselfs) and cumulative payoff (separately for each subject, and then entered into a group analysis based on SVO). These analyses found no significant difference in the average payoff or the cumulative payoff earned by prosocials and proselfs (Supplementary Fig. 6).

### Trial sequence flow

The game started with a graphical display for 3 s of human-like figures representing the subject (orange color) and located at the screen center, and figures (white color) representing five randomly selected candidate partners for a given trial and located around the subject figure. Next, only the figure representing the subject and one figure representing a randomly selected partner remained on the display screen for 3 s. Then, a text message reading, “It’s your turn” was displayed for 3 s. After that, a stake offer (e.g., JPY 100) was displayed with two buttons for a delay period of 4–6 s. At the end of the delay period, line boundaries were displayed around the buttons to signal subjects the time to make a choice. The left button had a capital letter “G” for the choice “Provide,” and the right button had a capital letter “N” for the choice “Not Provide.” The position of the buttons was fixed throughout the experiment (Fig. 1).

### Structural and functional MRI acquisition

Structural and functional brain images were acquired on a Siemens Trio TIM 3 T scanner at the Genetic Analysis and Brain Activity Imaging Laboratory (GBI) of Tamagawa University. High-resolution T1-weighted images were acquired for each subject with the following parameters: TR = 2000 ms, TE = 1.98 ms, flip angle = 10°, TI = 900 ms, 192 contiguous 1-mm sagittal slices, FOV = 256 mm, and voxel size = 1.0 × 1.0 × 1.0 mm. The parameters of the functional images were: TR = 2000 ms, TE = 25 ms, flip angle = 90°, FOV = 192 mm, voxel size = 3.0 × 3.0 × 5.0 mm, and 34 slices per brain volume.

### Statistical analysis of voxel-based morphometry

The structural T1 weighted images were processed and analyzed using SPM8 software (http://www.fil.ion.ucl.ac.uk/spm/, Welcome Department of Imaging Neuroscience Group, London, UK), and default parameters and routines as implemented by the VBM8 toolbox (http://dbm.neuro.uni-jena.de/vbm/). First, images were re-oriented to a canonical T1 template provided by SPM8 software. The images were then bias field corrected, tissue classified (gray matter, GM; white matter, WM; and cerebrospinal fluid, CSF), registered using linear (12-parameter affine) and non-linear transformations (warping) using a unified model^53^, and smoothed with a Gaussian kernel of 8 mm full width at half maximum (FWHM). We also applied masking with a threshold of 0.15 to restrict the search volume within GM.

The preprocessed images were entered into a multiple regression model in SPM8 to determine the brain regions which showed significant covariation with (1) SVO (prosocial and proself) and (2) cooperation rate of subjects as the first player in the PDG played in the fMRI experiment. We included age, gender, and total intracranial volume of each subject as covariates of no interest in the design matrix to regress out any effects attributable to them.

Based on our a priori hypotheses, region of interest (ROI) analyses were performed for the amygdala and the dorsolateral prefrontal cortex (DLPFC). Amygdala mask images, created separately for the left and right hemispheres, were constructed using the probabilistic cytoarchitectonic maps available in the SPM Anatomy Toolbox^54^ (SPM-AT). The final amygdala mask images were a summation of three amygdala voxel clusters^55^ (superficial, latero-basal, and centro-medial complex) available in SPM-AT.

The mask images of the left and right DLPFC (Supplementary Fig. 7) were created based on the results of a meta-analysis of 749 studies of functional neuroimaging findings and brain areas associated with the term “dorsolateral prefrontal” available in the database of Neurosynth (www.neurosynth.org) as of May 11, 2015. Neurosynth is “a platform for large-scale, automated synthesis of functional magnetic resonance imaging (fMRI) data” (text on the Neurosynth website). In this meta-analysis, we first identified the coordinates of the peak voxels in the left (z-score: 9.65; [x = −46, y = 34, z = 32]) and right (z-score: 12.89; [x = 42, y = 38, z = 32]) hemispheres that showed the highest z-scores in the results of a reverse inference analysis, which displays regions strongly associated with the term “dorsolateral prefrontal.” Because this method results in voxel clusters with asymmetrical sizes associated with the search term “dorsolateral prefrontal” in the left and right hemispheres, we first extracted only the voxels that showed contiguous localization with the peak voxels in the left and right hemispheres. Next, we created a final mask image of the DLPFC following a summation of all voxels in the left and right hemispheres using the Marsbar Toolbox (http://marsbar.sourceforge.net/) and used this DLPFC mask image in order to avoid biased results due to the use of mask images with different cluster sizes for each hemisphere. This procedure resulted in functional mask images of the left and right DLPFC with the following coordinate dimensions: x-min = 54, x-max = 18; y-min = 10, y-max = 60; z-min = −6, z-max = 48). The left DLPFC was created by adding a negative sign to the x-coordinates.

A threshold of P < 0.05, corrected for multiple comparisons separately for the amygdala (left and right hemispheres) and DLPFC (left and right hemispheres), was used. The SPM software was then used to extract the eigenvalues from the clusters of voxels showing significant correlations with SVO or cooperation rate. These values were entered into a simple linear regression analysis to search for a relationship between amygdala and DLPFC volume and subject’s cooperation rate in the PDG and tested with a Pearson correlation test.

### VBM results of whole-brain analysis

A whole-brain analysis was conducted to search for other brain regions showing possible correlation with SVO or cooperation rate using corrections for family-wise error (FWE) at a threshold of P < 0.05, and also using a more lenient threshold of P < 0.005, uncorrected, with voxel cluster size determined experimentally according to the SPM results table.

A positive correlation with SVO (regions larger in prosocials than in proselfs) was found in the left cerebellum (Supplementary Fig. 2 and Supplementary Table 1), whereas negative correlations with SVO (regions larger in proselfs than in prosocials) were found in the left inferior frontal gyrus, right DLPFC, right anterior cingulate, and left ventromedial orbitofrontal cortex (Supplementary Fig. 3 and Supplementary Table 2).

Positive correlations with cooperation rate (regions larger in highly cooperative subjects) were found in the left inferior temporal gyrus, right superior temporal sulcus, and right inferior temporal gyrus (Supplementary Fig. 4 and Supplementary Table 3). A negative correlation with cooperation rate (regions larger in less cooperative subjects) was found in the right cuneus (Supplementary Fig. 5 and Supplementary Table 4).

### Voxel-based morphometry results using anatomical mask images

In order to validate our analysis, which used mask images of the DLPFC and amygdala created based on probabilistic neuroimaging data, we also created mask images of the DLPFC and amygdala using anatomically defined data (Supplementary Fig. 8). The anatomical mask images of the amygdala (bilateral) and DLPFC (bilateral) were constructed from the Automated Anatomical Labeling (AAL) atlas^56^ as implemented in the WFU PickAtlas software^57^. Because the AAL atlas defines cortical regions of interest based on gyrus convolutions and anatomical landmarks, we chose the middle frontal gyrus as the lateral prefrontal region representative of the DLPFC.

This analysis confirmed the results presented in Fig. 1 and showed that the left amygdala was larger in prosocials than it was in proselfs (Supplementary Fig. 1a) and positively correlated with cooperation rate (Supplementary Fig. 1b). Similarly, the right DLPFC was larger in proselfs than it was in prosocials (Supplementary Fig. 1c), and the left DLPFC negatively correlated with cooperation rate (Supplementary Fig. 1d). Therefore, the consistency of these findings, using both probabilistic and anatomical mask images of the DLPFC and amygdala, suggest that our main findings were not an artefact of methodology, thereby validating our data analysis.

### Statistical analysis of fMRI data

The fMRI data of the subject in the first-player condition (two trial blocks, 15 trials each) were analyzed using SPM8. Each subject’s T1-weighted image was first re-oriented to a standard whole brain template available in SPM. All functional volumes were corrected for differences in slice time acquisition, realigned to the first volume, co-registered, spatially normalized to a standard echo planar imaging template included in the SPM software package, resliced into 2 × 2 × 2 mm voxels, and smoothed with an isotropic 6-mm FWHM Gaussian filter.

Subject-specific design matrices were created and contained the following regressors, whenever available, encoding the BOLD signal during the delay period (i.e., time lapse between the onset of the stake display and the go signal): (1) cooperative choice for JPY 100, (2) cooperative choice for JPY 200, (3) cooperative choice for JPY 400, (4) defection choice for JPY 100, (5) defection choice for JPY 200, (6) defection choice for JPY 400, (7) 6 nuisance regressors containing head movement displacement as estimated from the realignment procedure, and (8) nuisance regressors that encoded the reaction time (time lapse between the goal signal and a button press). These subject-specific design matrices were estimated, and the beta images for the first six regressors were entered into a multifactorial design with SVO (prosocial, proself), choice type (cooperation, defection), and stake size (JPY 100, JPY 200, JPY 400) as factors. We were interested in the BOLD signal of the brain regions for which we had a priori hypotheses (amygdala, DLPFC) and those that showed significant differences in GM volume between prosocial and proself subjects. Therefore, we performed an ROI analysis using as mask images the voxel clusters extracted at a statistical threshold of P < 0.001 (uncorrected) of the amygdala and DLPFC that survived a P < 0.05 corrected threshold in the VBM analysis.

Two types of analysis were performed using the identified anatomical clusters of the VBM analysis: (1) average BOLD signal as estimated from the beta images separately for prosocial and proself subjects, but regardless of stake size and choice type, and (2) average BOLD signal as estimated from the beta images separately for prosocial and proself subjects and choice type, but regardless of stake size. These analyses were conducted to test the null hypotheses that (1) no BOLD signal differences would be observed for the regions that had larger/smaller volumes in prosocial versus proself subjects, and (2) no BOLD signal-modulated activity would be observed for choice types (cooperation, defection). In this ROI analysis, only the voxel clusters identified within the anatomical mask images of the amygdala and DLPFC were analyzed with an FWE-corrected significance threshold of P < 0.05, unless otherwise specified. The BOLD signal and the time course of the signal were estimated using the RFX toolbox (http://rfxplot.sourceforge.net).

## Additional Information

**How to cite this article**: Fermin, A. S. R. *et al.* Representation of economic preferences in the structure and function of the amygdala and prefrontal cortex. *Sci. Rep.* **6**, 20982; doi: 10.1038/srep20982 (2016).

## Supplementary Material

Supplementary Information
