# Supplementary material for: Representation of economic preferences in the structure and function of the amygdala and prefrontal cortex
Source: Sci Rep. 2016 Feb 15;6:20982. doi: 10.1038/srep20982 (PMC4753461; doi:10.1038/srep20982)
Supplement: Supplementary Information [file srep20982-s1.doc]

**Scientific Reports**

**Supplementary Information**

**Representation of economic preferences in the structure and function of the amygdala and prefrontal cortex**

Alan S. R. Fermin^1^, Masamichi Sakagami^1^, Toko Kiyonari^2^, Yang Li^1^

Yoshie Matsumoto^1^, Toshio Yamagishi^3*^

^1^ Brain Science Institute, Tamagawa University

^2^ School of Social Informatics, Aoyama Gakuin University

^3^ Graduate School of International Corporate Strategy, Hitotsubashi University

* Corresponding author: Toshio Yamagishi, Graduate School of International Corporate Strategy, Hitotsubashi University, 2-1-2 Hitotsubashi, Tokyo 101-8439, Japan.

E-mail: yamagishitoshio@gmail.com

**Supplementary Figure 1**

**Supplementary Figure 2**

**Supplementary Figure 3**

**Supplementary Figure 4**

**Supplementary Figure 5**

**Supplementary Figure 6**

**Supplementary Figure 7**

**Supplementary Figure 8**

Supplementary figures’ legends

Supplementary Figure 1. Amygdala and dorsolateral prefrontal cortex (DLPFC) gray matter (GM) volume correlation with social value orientation (SVO) and cooperative behavior in the Prisoner’s Dilemma game (PDG) using anatomical mask images (see **Supplementary Methods**). **(a)** Left amygdala volume was significantly larger in prosocials than it was in proselfs (positive correlation with SVO, 46 voxels, x = -20, y = -7, z = -15, t = 2.32, P < 0.05 family-wise error (FWE) corrected). **(b)** Left amygdala volume positively correlated with cooperation rate (88 voxels, x = -24, y = 0, z = -21, t = 2.57, P < 0.05 FWE corrected). **(c)** Right DLPFC volume was significantly larger in proselfs than it was in prosocials (negative correlation with SVO, 97 voxels, x = 30, y = 41, z = 30, t = 3.28, P < 0.05 FWE corrected). **(d)** Left DLPFC was negatively correlated with cooperation rate (61 voxels, x = -32, y = 48, z = 33, t = 2.77, P < 0.05 FWE corrected).

Supplementary Figure 2. Whole-brain analysis showing positive correlations with social value orientation. This analysis revealed that the left cerebellum was larger in prosocials than it was in proselfs. The figures are displayed with a statistical threshold P < 0.001 and a voxel cluster size 58 as defined experimentally according to the SPM results table. This region survived a small volume correction with a 10-mm sphere radius and P < 0.05 family-wise error corrected. See **Supplementary Table 1** for statistical details.

Supplementary Figure 3. Whole-brain analysis showing negative correlations with social value orientation. This analysis revealed that the left inferior frontal gyrus, the right dorsolateral prefrontal cortex, and ventromedial prefrontal cortex were larger in proselfs than they were in prosocials. The figures are displayed with a statistical threshold of P < 0.001 and a voxel cluster size 58 as defined experimentally according to the SPM results table. These regions survived a small volume correction with a 10-mm sphere radius and P < 0.05 family-wise error corrected. See **Supplementary Table 2** for statistical details.

Supplementary Figure 4. Whole-brain analysis showing positive correlations with cooperation rate. This analysis revealed that the left inferior temporal gyrus, the right superior temporal sulcus, and right inferior temporal gyrus were larger in highly cooperative subjects. The figures are displayed with a statistical threshold of P < 0.001 and a voxel cluster size 58 as defined experimentally according to the SPM results table. The left inferior temporal gyrus survived a whole-brain analysis with a statistical threshold of P < 0.05 family-wise error corrected. The other regions survived a small volume correction with a 10-mm sphere radius and P < 0.05 family-wise error corrected. See **Supplementary Table 3** for statistical details.

Supplementary Figure 5. Whole-brain analysis showing negative correlations with cooperation rate. This analysis revealed that the right cuneus was larger in less cooperative subjects. The figures are displayed with a statistical threshold P < 0.001 and a voxel cluster size 58 as defined experimentally according to the SPM results table. The right cuneus region survived a small volume correction with a 10-mm sphere radius and P < 0.05 family-wise error corrected. See **Supplementary Table 4** for statistical details.

Supplementary Figure 6. Payoff analysis. **(a)** One-way ANOVA of the average payoff with social value orientation (SVO) as the main factor, F_1,976_ = 0.75, P = 0.3857. **(b)** One-way ANOVA of the average payoff, first calculated individually, and then entered into a group analysis with SVO as the main factor, F_1,31_ = 2.21, P = 0.1473. **(c)** One-way ANOVA of the cumulative payoff, first estimated individually, and then entered into a group analysis with SVO as the main factor, F_1,31_ = 2.96, P = 0.0955.

Supplementary Figure 7. Dorsolateral prefrontal cortex (DLPFC) as estimated by a meta-analysis available on the Neurosynth website (see **Supplementary Methods** for details). (**a**) Right DLPFC. The images were created to display the peak coordinate of the right DLPFC. (**b**) Left DLPFC. The images were created to display the peak coordinate of the left DLPFC. (**c**) Cortical surface view of the DLPFC. The voxels within the rectangles were extracted to create mask images of the DLPFC. The mask images were used to investigate the relationship between DLPFC structure and function and the results of this analysis are presented in the main paper. See Supplementary Information for details.

Supplementary Figure 8. 3D visualization of the of the middle frontal gyrus (MFG, top left and right panels) and the amygdala (bottom panel). Top left panel: left MFG in perspective (dark blue region). Top right panel: right MFG in perspective (purple region). Bottom panel: posterior view of the left (dark blue) and right (purple) amygdala. The structural anatomical mask images of the MFG and amygdala were created using the Automated Anatomical Labeling atlas (AAL, <http://qnl.bu.edu/obart/explore/AAL/>, Tzourio-Mazoyer et al., 2002) through the interface of WFU PickAtlas software (<http://fmri.wfubmc.edu/software/PickAtlas>, Maldjian et al., 2003). These anatomical mask images were used to confirm the results presented in the main paper.

| Supplementary Table 1. Whole-brain VBM results: areas with significant positive correlations with SVO | | | | | | | | | |
| --- | --- | --- | --- | --- | --- | --- | --- | --- | --- |
|  |  |  |  |  |  |  | P-value | | |
| Anatomical Location | MNI coordinates | | | Cluster size | t-value | z-value | Whole-brain uncorrected | Whole-brain FWE corrected | SVC FWE (10-voxel radius) |
|  |  |  |  |  |  |  |  |  |  |
|  | x | y | z |  |  |  |  |  |  |
| Left cerebellum | -20 | -55 | -60 | 317 | 4.45 | 3.38 | 0.001 |  | 0.001 |
|  |  |  |  |  |  |  |  |  |  |
|  |  |  |  |  |  |  |  |  |  |

Note. Small volume correction (SVC) was performed using a 10-mm sphere radius and P > 0.05 FWE corrected at the cluster level. VBM: voxel-based morphometry; SVO: social value orientation; MNI: Montreal Neurological Institute; FWE: family-wise error.

|  |  |  |  |  |  |  |  |  |  |
| --- | --- | --- | --- | --- | --- | --- | --- | --- | --- |
| Supplementary Table 2. Whole-brain VBM results: areas with significant negative correlations with SVO | | | | | | | | | |
|  |  |  |  |  |  |  | P-value | | |
| Anatomical Location | MNI coordinates | | | Cluster size | t-value | z-value | Whole-brain uncorrected | Whole-brain FWE corrected | SVC FWE (10-voxel radius) |
|  |  |  |  |  |  |  |  |  |  |
|  | x | y | z |  |  |  |  |  |  |
| Left inferior frontal gyrus | -39 | 30 | 3 | 111 | 4.52 | 3.84 | 0.001 |  | 0.008 |
| Right dorsolateral prefrontal cortex | 32 | 41 | 30 | 206 | 3.71 | 3.29 | 0.001 |  | 0.027 |
| Right anterior cingulate | 11 | 30 | -5 | 88 | 3.34 | 3.02 | 0.001 |  | 0.036 |
| Left ventromedial orbitofrontal cortex | -6 | 33 | -11 | 75 | 3.08 | 2.82 | 0.001 |  | 0.04 |
|  |  |  |  |  |  |  |  |  |  |

Note. Small volume correction (SVC) was performed using a 10 mm sphere radius and P > 0.05 FWE corrected at the cluster level. VBM: voxel-based morphometry; SVO: social value orientation; MNI: Montreal Neurological Institute; FWE: family-wise error.

|  |  |  |  |  |  |  |  |  |  |
| --- | --- | --- | --- | --- | --- | --- | --- | --- | --- |
| Supplementary Table 3. Whole-brain VBM results: areas with significant positive correlations with cooperation rate | | | | | | | | | |
|  |  |  |  |  |  |  | P-value | | |
| Anatomical Location | MNI coordinates | | | Cluster size | t-value | z-value | Whole-brain uncorrected | Whole-brain FWE corrected | SVC FWE (10-voxel radius) |
|  |  |  |  |  |  |  |  |  |  |
|  | x | y | z |  |  |  |  |  |  |
| Left inferior temporal gyrus | -41 | -13 | -26 | 595 | 6.35 | 4.89 | 0.001 | 0.001 |  |
| Right superior temporal sulcus | 51 | -7 | 10 | 259 | 4.02 | 3.51 | 0.001 |  | 0.02 |
| Right inferior temporal gyrus | 60 | -21 | -29 | 263 | 3.82 | 3.37 | 0.001 |  | 0.012 |
|  |  |  |  |  |  |  | 0.001 |  |  |
|  |  |  |  |  |  |  |  |  |  |

Note. Small volume correction (SVC) was performed using a 10 mm sphere radius and P > 0.05 FWE corrected at the cluster level. VBM: voxel-based morphometry; SVO: social value orientation; MNI: Montreal Neurological Institute; FWE: family-wise error.

|  |  |  |  |  |  |  |  |  |  |
| --- | --- | --- | --- | --- | --- | --- | --- | --- | --- |
| Supplementary Table 4. Whole-brain VBM results: areas with significant negative correlations with cooperation rate | | | | | | | | | |
|  |  |  |  |  |  |  | P-value | | |
| Anatomical Location | MNI coordinates | | | Cluster size | t-value | z-value | Whole-brain uncorrected | Whole-brain FWE corrected | SVC FWE (10-voxel radius) |
|  |  |  |  |  |  |  |  |  |  |
|  | x | y | z |  |  |  |  |  |  |
| Right cuneus | 15 | -84 | 37 | 261 | 4.91 | 4.09 | 0.001 |  | 0.002 |
|  |  |  |  |  |  |  |  |  |  |
|  |  |  |  |  |  |  |  |  |  |

Note. Small volume correction (SVC) was performed using a 10 mm sphere radius and P > 0.05 FWE corrected at the cluster level. VBM: voxel-based morphometry; SVO: social value orientation; MNI: Montreal Neurological Institute; FWE: family-wise error.
